# Case of Amyloid Degeneration of Liver, Spleen, and Kidneys

**Published:** 1878-04

**Authors:** W. T. Belfield


### CASE OF AMYLOID DEGENERATION OF LIVER, SPLEEN AND KIDNEYS.

By Dr. Belfield.

Jos. K——, aged 21, was admitted to the medical ward of the hospital, Sept. 28, 1877, and gave the following history.

There was no record of consumption in his family history. He had enjoyed robust health until June, 1874, when he suffered a six weeks' attack of bronchitis. After that time he remained entirely free from a cough until November, 1875, when he took cold again, and from that time was subject to a constant cough, at first dry and hacking, but subsequently accompanied with profuse expectoration, which had been yellow for almost a year prior to admission.

In December, 1876, he sprained himself one day while attempting to lift a very heavy weight. Next day his back was very stiff and sore, and became gradually worse. In March he noticed a lump in the course of the spine. At this time he had severe vomiting and purging for several days, but soon recovered his usual health. He noticed also a progressive emaciation which began about November, 1876.

In January, 1877, he had considerable pain in the right hypochondriac region, occurring at intervals for several weeks. From May to August, 1876, his right leg was much swollen, but neither tender nor painful. There was no œdema in the other leg nor elsewhere.

On admission he was much emaciated; his appetite and sleep were fair; his bowels moved, on the average, six times daily—the stools being of light yellow color; his tongue was clean and moist; his skin cool; pulse 100, rather small but not weak; temperature 98½. There was marked backward curvature of the spine, the most prominent point being the spinous process of the tenth dorsal vertebra. The skin was very translucent, the abdominal and thoracic cutaneous veins appearing quite large.

On examination there was found complete consolidation of the upper lobe of the right lung, except where a small cavity existed just below the clavicle; there was also infiltration at the left apex.

The hepatic dulness began at the fourth interspace and extended two inches below the level of the umbilicus. The free border of the liver, quite hard and rounded, could be traced on this level to the median line; thence the left lobe could be felt extending to the left hypochondriac region, its lower border on a level with the umbilicus. There were numerous tender spots over this hepatic area.

The spleen was not much enlarged vertically. Its anterior limit could not be well determined, owing to the intrusion of the enlarged liver. Its lower border could be felt just below the margin of the ribs.

The patient had been under treatment in the surgical wards for spinal caries during two months previous. On August 1 his urine had been of acid reaction, specific gravity 1013, and had contained five per cent. of albumen. No tube-casts were discovered. On admission to the medical wards (Sept. 28) the urine contained neither albumen nor casts.

His subsequent history was one of gradual decline. In November he had an obstinate diarrhœa; in December, an inflammation of the right knee. In January the urine contained ten per cent. of albumen and numerous hyaline and granular tube-casts. No record was made as to the quantity of urine passed in 24 hours.

Death occurred Feb. 9. An examination revealed a few ounces of clear serum in the peritoneal cavity. The liver was enormously enlarged, occupying a considerable portion of the abdominal cavity; yet this enlargement was symmetrical, so that the general outlines of the organ were not materially altered; its tissue was very smooth, firm, dense and elastic; the edges were thick and rounded. The cut surface was of pale red, waxy appearance—dry, hard and bloodless. The individual lobules were much enlarged and very distinctly outlined by white boundary-lines. A thin section was translucent. The kidneys were large, smooth, pale and firm, the enlargement being due to thickening of the cortical rather than of the medullary portion. The spleen was but little enlarged, but was firm, dense, and almost bloodless. But a small fragment of the right lung remained, the rest having been disorganized. The left lung presented a large cavity, numerous cheesy masses and many miliary tubercles. There was also extensive caries of the lower dorsal vertebræ. Microscopic sections exhibited admirably the iodine coloration. Entire lobules of the liver, excepting only the margins, presented the mahogany red color. The walls of the interlobular vessels were unusually thick, their calibre diminished. The liver cells were much enlarged, swollen, and rounded; they appeared to have no nuclei. In some instances there seemed to have been coalescence of two or more cells, from the obliteration of the intercellular substance. The cells at the periphery had, in many cases, undergone fatty degeneration. The cortical portion of the kidney was thickened and anæmic. The walls of the vessels were thickened; many of the vasa recta and nearly all of the malpighian bodies exhibited the mahogany-red color on the addition of iodine. The spleen was not much enlarged but was very dense and firm. Many of the malpighian bodies and vessels presented the characteristic color after staining with iodine.

